# Pulmonary epithelioid hemangioendothelioma accompanied by bilateral multiple calcified nodules in lung

**DOI:** 10.1186/1746-1596-6-21

**Published:** 2011-03-21

**Authors:** Xu Jinghong, Chen Lirong

**Affiliations:** 1 Department of Pathology, Second Affiliated Hospital, School of Medicine, Zhejiang University, Hangzhou, Zhejiang, 310009, PR China

## Abstract

Pulmonary epithelioid hemangioendothelioma (PEH) is a rare vascular tumor. It can present either as one solitary nodule or bilateral multiple nodules, usually without calcification. We describe here an unusual case of PEH in a 42-year-old female with a 6.0 cm dominant mass along with bilateral multiple calcified small nodules measuring 0.2-1.0 cm in diameter with a 25-year plus followup history. Overall histologic findings of the solitary tumor accorded with conventional PEH. While multiple calcified small nodules were composed predominantly of intra-alveolar homogeneously eosinophilic matrix, and only a few bland small cells were embedded in it. This lesion has never been reported in the literature. After comprehensive analysis of morphology, radiography, histochemistry, immunohistochemistry and differential diagnoses, PEH presenting multiple calcified small nodules was confirmed.

## Background

Epithelioid hemangioendothelioma (formerly known as intravascular bronchioalveolar tumor, IVBAT) is an uncommon tumor of vascular endothelial origin, with an intermediate course between hemangioma and conventional angiosarcoma[1-3]. PEH typically occurs as bilateral multiple nodules among young women[1,4]. But some cases also develop as a solitary lung nodule or a single cavitary nodule[1,5]. We describe here a case of PEH presenting as a 6.0 cm dominant mass along with multiple calcified small nodules in lung. To our knowledge, this type of PEH manifestation has not been reported in the literature before.

## Case presentation

**Figure 1:**
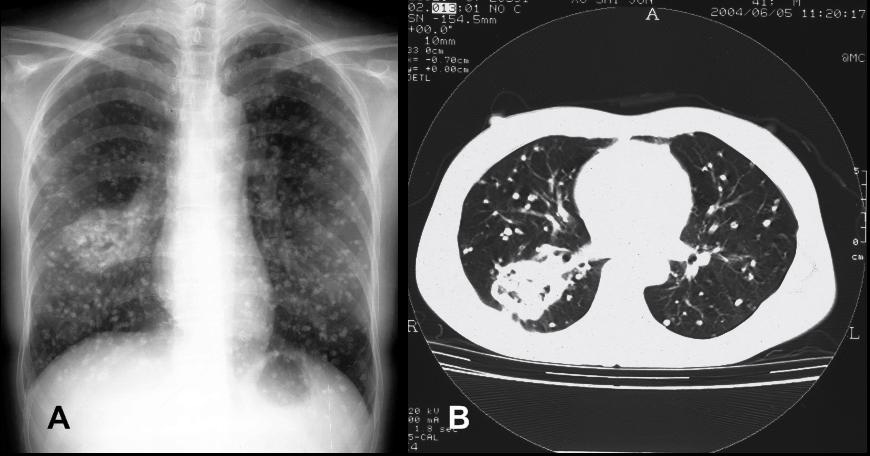
**Radiographs of chest**. Chest radiograph (A) and computerized tomograph (B) show an approximately 6.0 cm sized well-demarcated mass in the right lung and multiple calcified small nodules in both lungs.

In 1982, a 20-year-old, non-smoking woman was incidentally found to have an abnormal chest X-ray during a routine medical examination. The X-ray radiograph showed diffusely scattered, high-density small nodular opacities in all the lung fields. Routine laboratory data were within normal limits at the time. She had no significant complaints, past illnesses, or family history. The multiple high-density small nodular opacities were slowly increasing in number and size in the serial follow-up chest radiographs. Twenty years later (May 2002), a follow-up X-ray film showed one approximately 3.0 cm sized, well-defined mass in the middle field of right lung. Because of the absence of symptoms, she had always rejected any medical interventions. In July 2004, she began to cough with sputum. A plain chest radiograph (Figure 1A)and computerized tomograph (CT) (Figure 1B) revealed the mass to be increased to 6.0 cm along with more calcified small nodules (all smaller than 1.0 cm). Physical examination and laboratory findings were still not unremarkable. In September 2004, the patient underwent a lobectomy of the right lower lobe.

**Figure 2:**
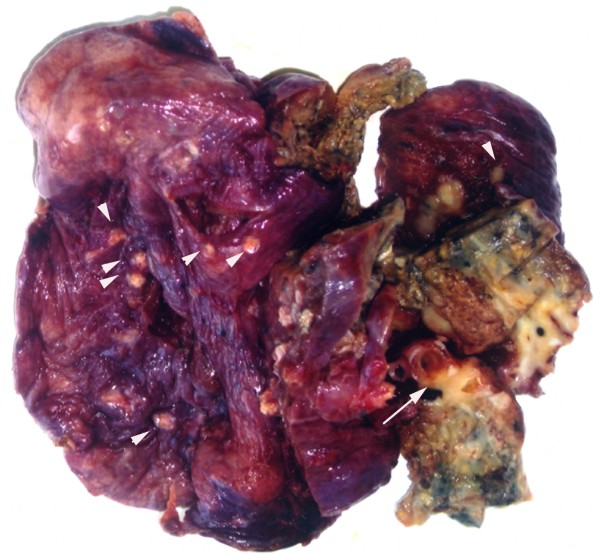
**Macroscopic aspect**. The cut surface reveals bronchial invasion of the tumor (long arrow), and multiple small nodules under the visceral pleura (arrowhead).

Macroscopically, the specimen measured 12.0 × 10.0 × 4.0 cm in size and was mostly replaced by a well-circumscribed, unencapsulated mass, up to 6.0 × 5.0 × 4.0 cm. The cut surface of the mass was whitish to brownish in color and solid, but focal cystic areas filled with necrotic material were noted. The tumor surrounded the bronchus and invaded its wall. In addition, more than 50 calcified small nodules were noted, measuring from 0.2 cm to 1.0 cm in diameter. The nodules were scattered throughout the whole lobe including within the main mass itself (Figure 2).

**Figure 3:**
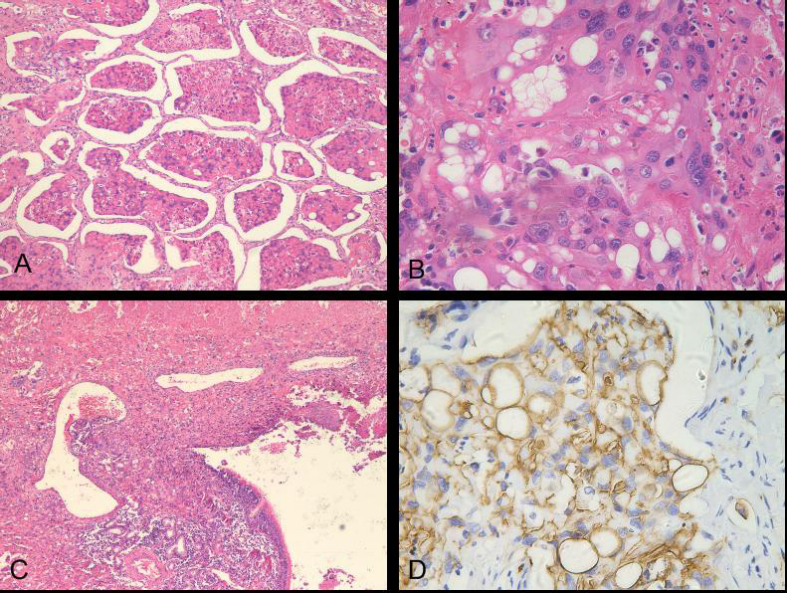
**Histologic findings of the solitary mass**. (A) At the periphery, tumor exhibited micropolypoid growth, and central coagulative necrosis existed in the cell cluster (H and E; original magnification ×100). (B) Several tumor cells showed marked cytologic atypia with large hyperchromatic nuclei, vesicular chromatin and prominent eosinophilic nucleoli (H and E; original magnification ×400). (C) The tumor showed extensive necrosis (upper) and invaded to bronchial mucosa and blood vessel (H and E; original magnification ×40). (D) Immunostaining for CD34 revealed strong and diffuse positivity of epithelioid cells and prominent cytoplasmic vacuoles and lumens (original magnification ×400).

Histologically, the dominant large mass was composed of small nests, cords and strands of epithelioid cells embedded in a highly eosinophilic stroma. Extensive tumor cell necrosis and stromal sclerosis were present. At the periphery of the tumor, micronests with central coagulative necrosis extended to alveolar spaces in a contiguous, micropolypoid fashion and through pores of Kohn in alveolar walls (Figure 3A). The epithelioid cells were mildly atypical, with polygonal to plump, abundant eosinophilic cytoplasm, irregular round nuclei, coarsely granular chromatin, and occasional nucleoli. More severe atypical cytologic changes were also seen, including larger nuclei, prominent eosinophilic nucleoli and intranuclear cytoplasmic inclusions (Figure 3B). Many tumor cells showed characteristic intracytoplasmic vacuoles or lumens, some of which containing erythrocytes or fibrin. The intercellular stroma consisted of abundant hyalinized matrix with focal mucinous degeneration. Areas of sheet-like eosinophilic matrix with a few or single tumor cells were observed. The alveolar septal outline was vaguely seen. The aggressive behavior of the tumor was demonstrated with vascular invasion, bronchiolar and bronchial invasion (Figure 3C), endobronchiolar spread, and metastasis to two hilar lymph nodes.

**Figure 4:**
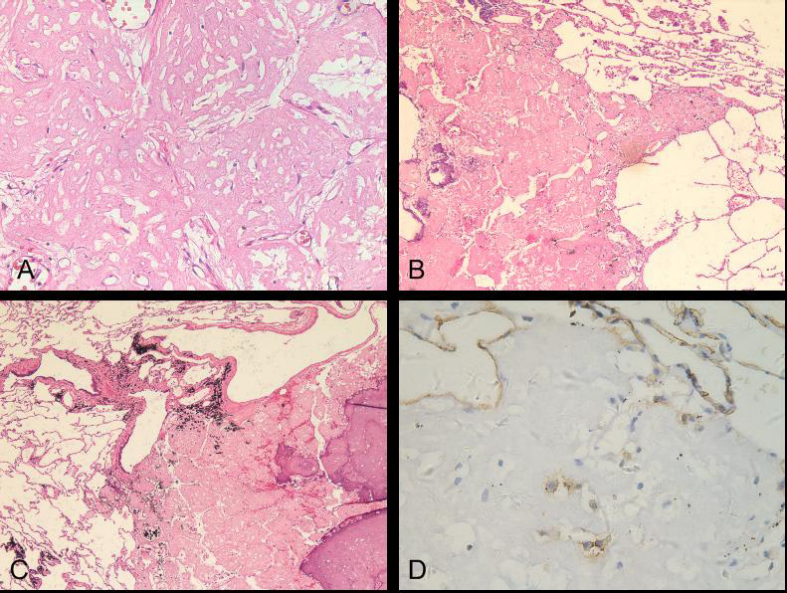
**Histologic findings of the multiple small nodules**. (A) Eosinophilic substances were filled in pulmonary alveolar space; Cells in the nodule were small and moderate (H and E; original magnification ×200). (B) Border of the small nodule with partial calcification was not smooth and extended to neighbor airspaces (H and E; original magnification ×40). (C) The small nodule was located with the bronchiolar and vascular walls (H and E; original magnification ×40). (D) Individual small cells showed positive immunoreactivity for CD31 (original magnification ×400).

On the other hand, the multiple small nodules were composed predominantly of intra-alveolar, homogeneously eosinophilic matrix with a few small cells embedded. These cells were cytologically not as atypical as the cells in the main mass (Figure 4A). A few scattered inflammatory cells infiltrated throughout the lesion. At low magnification, the alveolar outlines could be basically identified. Some alveolar capillaries in interalveolar septum also existed and were dilated. Partial, complete calcification and ossification of small nodules were noted. The lesion border was not smooth and extended to adjacent alveolar spaces (Figure 4B). Most of small nodules were closely related to bronchioles, bronchi and blood vessels (Figure 4C). Some joined to the bronchiolar or bronchial walls; some adhered to the vascular walls.

Histochemically, alvelolar reticular fibers highlighted with a reticulin stain were preserved at the periphery of the tumor and partially existed in small nodules. Abundant eosinophilic matrix was depicted light green color with a modified Masson trichrome stain and red color with a Van-Gieson (VG) stain. Congo Red and Crystal violet stains were negative in both the main mass and small nodules.

Immunohistochemically, in both the main tumor mass and the small nodules, cells were strongly and diffusely positive for vimentin, and the acellular matrix was positive for factor VIII-related antigen. CD34 and CD31 were strongly positive in the main tumor mass (Figure 3D) and showed scattered positivity in the small nodules (Figure 4D). The cells in all lesions were negative for cytokeratin7, cytokeratin17, cytokeratin18, cytokeratin19, cytokeratin20, S-100 protein, smooth muscle actin, epithelial membrane antigen and thyroid transcription factor-1.

## Discussion

We consider our case to be a PEH with a long standing, relatively benign clinical history and with a unique histological and radiological presentation.

In our case, the main mass showed a typical PEH morphology. But the small nodules appeared uniformly hypocellular and consisted mainly of eosinophilic matrix, which occupied alveolar spaces and was indicated to collagen by modified Masson trichrome stain and VG. However, result of the immunostains did confirm the endothelial cell nature of the rare cells even though the epithelioid morphology was somewhat lacking. The eosinophilic matrix in the satellite nodules and the way it filled and extended to adjacent alveolar spaces was the same as the main mass and fitted the description of PEH in that the neoplastic micronest spread through alveolar pores without destruction of the alveolar epithelium and interalveolar septum. Reticulin stain confirmed the preservation of the alveolar reticular network in both the multiple small nodules and the main mass. In addition, the relationship of the multiple small nodules to bronchioles, bronchi and vessels bore some resemblance to the invasion of bronchioles, bronchi and vessels in the main tumor mass, which was one of the PEH features described on CT by Luburich P et al [6].

Radiographically, the most characteristic presentation of PEH is bilateral multiple nodular opacities up to 2.0 cm in diameter, with well- or ill-defined margins [1-3],10-19% develop as a solitary nodule [1]. They show little or no growth on serial radiographs and are usually found in relation to small and medium sized vessels and bronchi [6]. While histological calcification is common, radiologic calcification is not [2,6]. In long-standing cases or after treatment, extensive calcification of the nodules can be seen [6-8]. An interesting PEH case has been described by Ledson et al [7]. A woman presented multiple irregular, unequal, uniformly distributed, non-calcified nodular shadowing in bilateral lungs on chest radiograph. The size of nodules was up to 15 mm. PEH was confirmed by open lung biopsy. Due to the hypertrophic pulmonary osteoarthropathy, she took indomethacin and azathioprine. After 8 years, radiograph showed increasing calcified nodules in all areas. After 7 years again, the overall number of calcified nodules remained unchanged, but their size had decreased to a maximum of 12 mm.

The differential diagnosis of calcified nodules includes other diseases with pulmonary calcification showing multiple small nodules, including pulmonary alveolar microlithiasis (PAM), amyloidosis, pulmonary hyalinizing granuloma (PHG) and multiple calcifying fibrous tumors (CFT). PAM is a rare disorder of unknown etiology. Radiological features reveal bilateral, diffusely scattered micronodular shadows of calcific densities, which are similar to our case. However, microscopic studies find intra-alveolar and interstitial deposits of concentrically laminated calcified bodies [9].

Amyloidosis also presents an amorphous eosinophilic material and secondary calcification in lung parenchyma and airways [9]. In our case, negative Congo Red and crystal violet stains helped to exclude the diagnosis. PHG is a rare fibrosing lesion, sometimes presenting with multiple calcified nodules. It also occurs in middle-aged persons with no clinical symptoms. But its characteristic histological appearance is central whorled deposits of lamellar hyalinized collagen with nonspecific inflammatory infiltration[10] which this case did not have. Multiple CFT is a benign lesion characterized histologically by hypocellular, dense, hyalinized collagenous tissue with psammomatous and/or dystrophic calcifications and patchy lymphoplasmacytic infiltrates [11]. Immunohistochemical staining reveals that most spindle cells are positive for vimentin, CD34 and factor XIIIa [11]. In our case, neither psammoma bodies nor patchy lymphoplasmacytic infiltrates were found. And up to date, few cases have been described in the pleura, but none in the lung [11].

## Conclusions

We describe an unusual case of pulmonary epithelioid hemangioendothelioma (PEH) presenting as one solitary mass along with bilateral multiple calcified small nodules, which showed intra-alveolar homogeneously eosinophilic matrix with a few bland small cells embedded. To our knowledge, the extremely long history, the unique radiological findings that were confirmed by histology and immunohistochemistry has not been reported yet. Our case illustrates the amazing indolent nature a pulmonary epithelioid hemangioendothelioma can have and the differential diagnoses that have to be kept in mind when handling such a case.

## Consent

Written informed consent was obtained from the patient for publication of this case report and any accompanying images. A copy of the written consent is available for review by the Editor-in-Chief of Diagnostic Pathology.

## Competing interests

The authors declare that they have no competing interests.

## Authors' contributions

XJ participated in conducting pathological examinations and wrote the manuscript. CL analyzed the case and prepared the manuscript. All authors have read and approved the final manuscript.
